# Experimental Evidence for Edge‐Mediated Microclimatic Refuge in a Southern Range Margin Population of *Silene regia*

**DOI:** 10.1002/ece3.73507

**Published:** 2026-04-16

**Authors:** Megan Nicole Brooks, Adamina Bilbrey, Heather Bowman Cutway, Nicholas Green, Mario Bretfeld

**Affiliations:** ^1^ Department of Ecology, Evolution, and Organismal Biology Kennesaw State University Kennesaw Georgia USA; ^2^ College of Liberal Arts and Sciences Mercer University Macon Georgia USA

## Abstract

Due to widespread grassland loss, native perennial grassland species such as Royal catchfly ( *Silene regia* ) are today restricted to fragmented habitats such as roadsides and powerline rights‐of‐way. In Georgia (United States), the species is listed as critically imperiled, and reintroduction efforts have had limited success, highlighting the need to identify environmental factors supporting its persistence at the southern margin of its range. This study investigated whether microclimatic and edaphic factors associated with a forest–grassland edge influence the abundance, growth, and fecundity of *S. regia* in Georgia's (US) last confirmed wild population. We monitored 59 and 81 individual plants across the 2023 and 2024 growing seasons, respectively, in a managed powerline right‐of‐way where an open grassland transitions sharply into a forest. Data were collected on spatial distribution, plant height, flowering, seed capsule production, soil nutrients, and microclimate (air temperature and soil moisture). The 2024 season followed a right‐of‐way widening, which shifted the forest–grassland edge by approximately 2.8 m. Individuals within a 2 m wide band along the forest–grassland edge exhibited significantly greater height, flowering, and seed capsule production compared with those in the open grassland or forest interior. The location of maximum growth and reproduction shifted between years in accordance with the physical movement of the edge, providing experimental evidence for an edge effect. Soil nutrient composition did not differ significantly across the site, but daily maximum air temperature was significantly higher in the open grassland than along the edge or within the forest. Our results suggest that partial shading along forest–grassland boundaries mitigates heat stress and enhances resource allocation to growth and reproduction in *S. regia* , providing a climatic and competitive refuge at the southern boundary of its range.

## Graphical abstract

We examined spatial distribution, growth, and reproductive output of the grassland perennial *Silene regia* at the southern edge of its geographic range in Georgia, USA, across a forest‐grassland boundary. Individuals located along the edge exhibited greater growth and fecundity than those in adjacent open grassland or forest, a pattern that shifted following experimental movement of the edge and coincided with reduced temperature maxima. These results indicate that forest‐grassland edges can function as microclimatic refugia that enhance population performance at range margins.

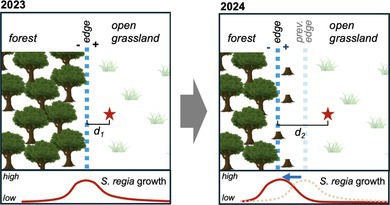

## 1 Introduction

In most of the 20th century, native grassland and prairie ecosystems declined due to wildfire suppression, the conversion of habitat for agricultural and developmental purposes, and diminishing populations of essential pollinators and grazing species (DeSantis et al. 2011; Wagle and Gowda 2018; Lark 2020). These ecosystems are now confined to fragmented areas which experience lower biodiversity and altered ecosystem dynamics (DeSantis et al. 2011; Fritz and Nilsson 1994; Eric S. Menges 1991). Global climate change is predicted to further threaten these already periled ecosystems as temperatures, precipitation, and seasonal patterns become more unpredictable, potentially altering community composition and biodiversity (Bellard et al. 2012; Hooper et al. 2005, 2012). In more recent decades, there have been increasing conservation efforts to safeguard and restore these ecosystems via habitat protection as well as the re‐introduction of natural fire regimes and native species (Bond and Keeley 2005; Trowbridge et al. 2017). This study seeks to aid in these efforts by increasing the knowledge of regional ecological information surrounding a state‐endangered Georgia (US) native grassland species, *Silene regia* , commonly known as the ‘royal catchfly’.

*Silene regia* is an herbaceous perennial native to the midwestern and southeastern grassland ecosystems of the United States. The species exhibits perfect flowers, is supported via a strong taproot, and prefers shallow, calcareous soil (King 1981). Pollination occurs mainly by the ruby‐throated hummingbird ( *Archilochus colubris* ), and reproduction is carried out solely through seed production and dispersal, as it does not propagate clonally. Although historically distributed in twelve states spanning from the Midwest into the southeast, *S. regia* is currently ranked as vulnerable (S3) or higher in the entirety of its native range (NatureServe 2025). Today, due to the loss of 95% of its habitat, the species is typically confined to small patches of remnant grassland habitats such as roadsides and cemeteries (Dolan 1995), posing a challenge for conservation. Remaining fragmented populations are more susceptible to reduced genetic variation, which can lead to inbreeding depression and a reduction in viability of offspring (Eric S. Menges 1995; Aguilar et al. 2019) and display greater sensitivity to environmental stochasticity (Eric S. Menges 1991). Although established *S. regia* plants are long lived, recruitment of viable seedlings is necessary for continued growth and expansion of a population (Eric S. Menges 1995). Besides habitat loss and fragmentation, the lack of an appropriate fire management practice has been posited as the most prevalent threat to these populations (Menges and Dolan 1998). Without fire, *S. regia* populations have been shown to shift from high growth and fecundity characteristic of an open habitat species towards a closed habitat strategy, with shifts towards survival‐oriented traits (Menges and Dolan 1998). Kull (2020) notes that the downward trend in *S. regia* populations at one site in Missouri is most likely directly tied to an increased fire return interval from historically 4–8 to nearly 10 years.

Previous studies on the ecology and life history for this species have focused mainly on Midwest populations and may not accurately represent the conservation need of southern populations, where extreme temperatures and prolonged periods of drought are predicted to intensify in coming decades (Anandhi and Bentley 2018). Attempts to outplant *S. regia* to new locations within Georgia (US) have had only limited success (Georgia Plant Conservation Alliance; *pers. communication*). We aimed to identify local environmental factors that contribute to the persistence of the last confirmed wild population of *S. regia* in Georgia (US) by assessing local ecological variables as well as plant growth and reproductive output. Our study was further motivated by anecdotal reports from conservation partners noting that individual plants that were located closer to adjacent trees exhibited more vigorous growth. This observation is consistent with findings from a Midwestern population at Wilson's Creek Battlefield in Missouri, where Kull (2020) reported that surveyors in 1989 observed all populations within the park occurring along successional edges between early‐stage grassland and later‐stage shrubland or forests.

Previous research has shown that fragmented populations may benefit from edge dynamics in which beneficial conditions (i.e., “complementary” resources) from two adjacent areas may be utilized by the local species (Ries et al. 2004, 2017; Cadenasso et al. 1997). In populations where these so‐called “edge effects” exist, one could expect population abundances to increase at the transition area relative to the interior of either ecosystem (Ries et al. 2004, 2017). In comparisons of species richness along forest interior and edges to grassland interiors and edges in northeastern France, Burst et al. (2017) found that total species richness was significantly higher at the grassland edge and decreased towards the core of either interior. Complementary resources along a forest‐grassland transition area include increased shade from trees, leading to reduced air and soil temperature as well as increased humidity (Cadenasso et al. 1997). Similar abiotic conditions have been well documented in a variety of ecosystems where forest canopies have been observed to function as temperature buffers (De Frenne et al. 2019). In a survey of 98 sites across five continents, De Frenne et al. (2019) found that average and maximum temperatures underneath the forest canopy were cooler than temperatures of the surrounding area by 1.7°C ± 0.3°C and 4.1°C ± 0.5°C, respectively. In a study conducted in northwestern U.S. forest stands, it was found that maximum temperatures can be as much at 5.3°C lower under forest canopy areas than areas without canopy cover (Davis et al. 2019). Species with narrow climatic associations are under the highest risk of extirpation and extinction, and microclimatic refuge provided by spatial and topographical variations has been observed to reduce the extirpation risk by 22% in threatened plant species that are adversely affected by warming trends (Lenoir et al. 2017; Suggitt et al. 2018). Grassland species such as *S. regia* could benefit from these stress reducing conditions along an edge area, especially when growing in the southern‐most range of its geographic distribution.

Edge effects caused by fragmentation in landscapes have also been examined in the context of soil nutrient and moisture characteristics (Piessens et al. 2006; Ruwanza 2019). In Hungary, Erdős et al. (2013, 2019) found that soil moisture along forest‐grassland boundaries was significantly higher than open grassland while being lower than fully forested areas. The same study found that mean soil temperature values for the edge area were intermediate between the forest and grassland. These differences in soil condition were correlated with higher species richness and Shannon‐diversity index for plant species, with some species observed to be edge‐specialists (Erdős et al. 2013). The creation of a favorable soil microclimate along edges may result in higher litter decomposition and increased nutrient availability, thus promoting growth for edge adapted plants (Ruwanza 2019). Terrestrial ecosystems, such as grasslands, are frequently nutrient limited (Fay et al. 2015). Nitrogen and phosphorus are two of the most common limiting elements considered in most ecosystems, including in grasslands (Fisher et al. 2012). A potential increase in the concentration of soil nutrients or the presence of more favorable soil moisture and temperature conditions along an edge area may provide an advantage for perennial grassland species such as *S. regia* .

Edge dynamics, such as those previously mentioned, have not been tested for their potential benefits to *S. regia* , a species that is known to thrive in open, sunny habitats. Given that *S. regia* occurs at the southernmost extent of its range in Georgia (US) and that regional climate is projected to become warmer and drier, edge refugia may offer valuable habitat for this vulnerable species throughout its range. We monitored growth and fecundity of a remnant *S. regia* population in Georgia (US) located along a roadside right‐of‐way over the 2023 and 2024 growing seasons. We hypothesized that there will be a higher abundance of individuals along the forest‐grassland edge compared to the adjacent forested or open grassland areas. We further hypothesized that plants occurring along the edge will exhibit greater height, increased flowering, and higher rates of successful seed capsule production. Finally, we hypothesized that these edge‐associated benefits will shift in response to altered light and microclimatic conditions following selective tree removal in 2024 to widen the right‐of‐way. Specifically, we expected the zone of enhanced growth and fecundity to track the physical shift of the forest edge. Our results will aid conservation efforts by informing conservation partners about optimum regional translocation strategies for this species.

## 2 Materials and Methods

### 2.1 Site Description

This study took place in Dade County, located in northwestern Georgia, USA, a region characterized by a humid subtropical climate. Summers are typically hot, with average temperatures ranging from 21°C to 30°C, while winters are mild, with average temperatures between 2°C and 11°C (National Weather Service 2024). Annual precipitation averages between 137 and 157 cm, most of which is rainfall (National Weather Service 2024). The terrain is predominantly comprised of limestone, shale, and sandstone (Lawton et al. 1976). The sampled population is situated within an approximately 70 × 30 m section of a state highway roadside right‐of‐way, which supports numerous grassland indicator species and, as of 2017, represents the last confirmed wild population of *S. regia* in Georgia (Georgia Department of Natural Resources 2025). Over the past two decades, the site has been regularly monitored and actively managed by the local landowner in collaboration with Georgia Power, the Georgia Plant Conservation Alliance (GPCA) and its affiliated safeguarding partners. Management includes regulated mowing and prescribed burning.

The roadside right‐of‐way is characterized by a field resembling an open grassland that is bordering an adjacent forested area characterized by a blend of coniferous and deciduous trees, with a distinct edge delineating the transition between the two areas. *S. regia* individuals are present in all three areas within the site: the full shade forested area, the open grassland area, and the edge transition between the two. Because the trees are located to the west of the open grassland area, individuals close to the edge are subjected to afternoon shade. Local native grassland species include prairie rosinweed ( *Silphium terebinthinaceum* ), browneyed susan ( *Rudbeckia triloba* ), tall thimbleweed ( *Anemone virginiana* ), and partridge pea ( *Chamaecrista fasciculata* ). The forested area of the site is dominated by eastern redcedar ( *Juniperus virginiana* ), red oak ( *Quercus rubra* ), and blackjack oak ( *Quercus marilandica* ).

In February 2023 and as part of the management plan of this site, a controlled burn was conducted by the Georgia Forestry Commission. The subsequent ease of locating *S. regia* individuals provided an excellent opportunity to examine growth and reproduction of the population present. In early 2024, the right‐of‐way was widened by Georgia Power to safely accommodate the utility lines. Widening was performed using low‐impact practices to minimize damage to plants and soil and resulted in the forest‐grassland edge being pushed back by an average of 2.76 m. This manipulation and resulting shift in the edge created a quasi‐experimental, before‐after comparison between 2023 and 2024, allowing us to strengthen causal inference.

### 2.2 Field Sampling

**FIGURE 1:**
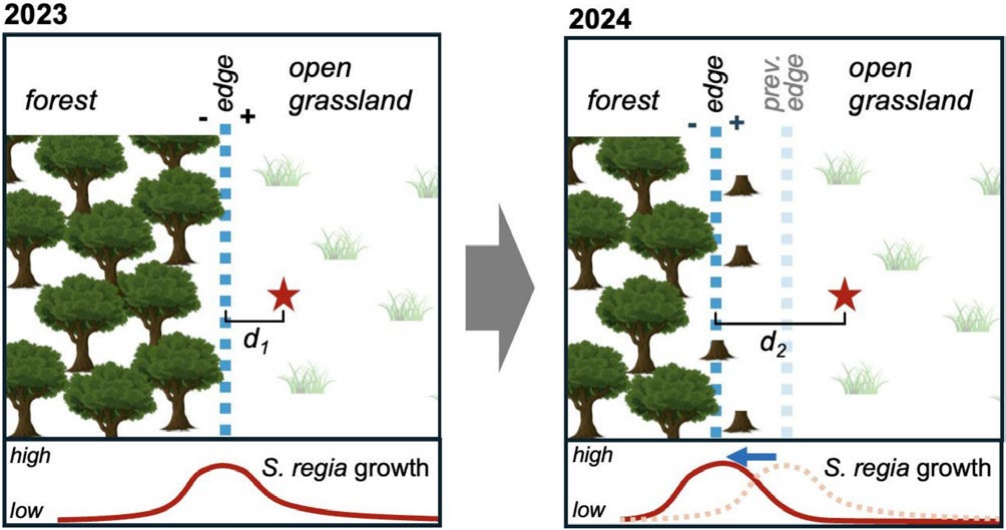
Schematic of sampling technique on site and hypothesized growth trend. The red star represents an individual *S. regia* plant. Its distance to the edge line transect (blue dashed line) is indicated by *d*_1_ in 2023 and *d*_2_ (after widening of the right‐of‐way) in 2024.

In April 2023, we flagged and assigned unique identifiers to 59 individual *S. regia* plants. For each individual in the site, we recorded the distance *d*_1_ relative to a 60‐m tape laid along the forest‐to‐grassland edge (Figure 1), with negative values indicating plants growing on the forest side and positive values indicating plants growing on the grassland side. From May to September 2023, regular visits (every 2 weeks for a total of 10 visits) were made to the site until mature seed capsules were formed. In the following year and following the widening of the right‐of‐way, surveys were conducted using the same sampling design and biweekly visitation schedule as in 2023. In 2024, 44 of the flagged 59 individuals re‐emerged and 37 new plants were discovered, for a total of 81 *S. regia* plants that were monitored in 2024. Similar to the previous year, the relative distance *d*_2_ of each individual was recorded, now with respect to the shifted edge, and all plants were monitored biweekly over the 2024 growing season (May to September; 10 visits). During each site visit, we recorded plant status (dead/alive), height of the tallest stem, and counted the total number of mature flowers and ripe seed capsules for each individual.

Abiotic data collection in 2023 comprised the collection of 17 soil samples across the three areas. Soil samples were obtained on May 5 every 6 m along three 36 m transects running parallel to the forest‐to‐grassland edge. One transect was placed within the forest (6 samples), one along the edge (6 samples), and one in the grassland (5 samples), providing systematic coverage of the study area. To obtain each sample, vegetation and leaf litter were brushed away from the surface before collecting approximately 450 g of soil from a depth up to 6 cm. All samples were then sent to the University of Georgia Extension Agricultural and Environmental Services Labs (Athens, GA) for analysis of pH, lime buffer capacity (LBC), organic matter (OM), cation exchange capacity (CEC), phosphorus (P), potassium (K), calcium (Ca), magnesium (Mg), zinc (Zn), manganese (Mn), and other inorganic content. Briefly, the Meclich‐1 soil extraction method was used in mineral and nutrient content testing, and LBC and pH were determined using LabFit AS‐3000 pH equipment via titration.

In 2024, we installed a battery‐powered data logger (CR1000; Campbell Scientific, Logan UT) paired with a multiplexer (AM16/32; Campbell Scientific, Logan UT), nine soil moisture sensors (ECH2O EC‐5; Meter Group, Pullman WA), and nine air temperature sensors (model 107 & 108; Campbell Scientific, Local UT) to obtain a high temporal resolution of soil moisture as well as other microclimatic dynamics. Three soil moisture and three air temperature sensors with radiation shields were installed at least 3 m into the forest; an additional set of three soil and three air sensors were installed along the edge, and the final set was installed at least 3 m from the edge into the grassland area. Soil moisture was measured in the top 5 cm of the soil. Air temperature was measured at 1.5 m height, reaching slightly above the vegetation in the grassland at time of installation. Data were collected every 30 s, averaged, and logged every 15 min from July 29 to October 4, 2024. Although data do not encompass the entire growing season, we assume that the observed trends are representative of patterns across the full season.

### 2.3 Data Analysis

For our analyses, plants were categorized into three groups based on their distance (*d*_*i*_) from the edge. Individuals located between −4 m and −1 m (i.e., up to 4 m into the forest) were categorized as “forest”; plants within −1 m to +1 m were considered to be part of the “edge” zone; and plants located at +1 m or farther (i.e., at least 1 m into the grassland, up 9 m) were considered to be part of the “grassland” (Figure 1). Note that due to the shifting edge, the farthest distances at which plants were found within the forest were −4 m in 2023 and −2 m in 2024, while the farthest into the grassland were 5 and 9 m in 2023 and 2024, respectively.

To assess whether more individuals grow along the edge, we used only 2023 data. The 2024 data were excluded from the analysis because the time since right‐of‐way widening was likely too short for seed‐based establishment. The 37 new individuals observed in year two were likely either previously undetected or reemerged from a vegetative state rather than having germinated from seed, based on their large sizes throughout.

Given the 2‐m wide edge and the maximum distances at which individuals were located in respect to a 60‐m‐long tape, we first estimated the approximate area of each of the three zones: $\left|-1\;m-\left(-4\;m\right)\right|\times 60\;m=180\;m^{2}$ (forest); $\left|-1\;m-1\;m\right|\times 60\;m=120\;m^{2}$ (edge); $\left|1\;m-5\;m\right|\times 60\;m=240\;m^{2}$ (grassland). To evaluate whether plant abundance was disproportionately concentrated in the edge zone, we compared observed counts to area‐proportional expected counts assuming equal distribution. A Chi‐squared goodness‐of‐fit test was used to assess deviations from uniform density across the site. Because we have no replications of our site, this test serves a descriptive rather than inferential role, providing insight into spatial patterns within the bounds of our study site alone.

To test for potential edge‐related trends in plant growth, part of our second hypothesis, we fit plant height data to a non‐linear Gompertz curve model for each of the two monitored seasons.

$$y=\text{Asym}\times \exp\left(-b_{2}\times b_{3}^{x}\right)$$

where *y* is the predicted plant height (cm) on day *x*, Asym is the asymptotic maximum height, and *b*_2_ and *b*_3_ are curve‐shaping parameters related to growth rate and curve steepness, respectively. This approach accounts for repeated measurements of individual plants within each growing season and enabled us to estimate expected plant height over time across the population. The residuals from that model (i.e., the deviation of each plant's observed growth from the predicted average growth) were then tested for trends along each individual plant's distance from the edge (*d*_*i*_) following the edge effect model framework outlined by Ries et al. (2004). Specifically, the framework predicts that when there are complementary resources present along the boundary of two habitats, the response of the organism of study will display a peak along such an edge. Accordingly, we applied an ordered polynomial contrast approach to test for statistically significant peaks in residual plant height across 1‐m‐wide transects arranged parallel to the forest–grassland edge, after accounting for time‐based growth trends using the model described above. To visualize significant peaks, we overlaid model residuals with best‐fitting polynomial regression lines, up to the third‐degree polynomial (i.e., cubic). Higher‐order terms (fourth‐degree and above) were omitted as they were either statistically non‐significant or offered minimal improvement while increasing the risk of overfitting. Additionally, to assess whether residuals deviated significantly from expectation at specific distances from the edge, we fit a linear model without an intercept. This model estimated the mean residual value for each 1‐m‐wide bin, allowing us to identify bins with significantly higher or lower than expected growth.

To test for potential edge‐related trends in flower and seed capsule counts, part of our second hypothesis, we fit a negative binomial generalized linear model (GLM) using plant height as the predictor variable given that plant size is a known factor influencing reproductive output (Liu and Pennings 2019). We chose a negative binomial model due to the high proportion of samples with zero flowers or fruit. Similar to the plant growth model, we assessed residuals from this model for significant peaks using polynomial contrasts and then fit a linear model without an intercept to estimate mean residuals per bin. Analyses were performed independently for each growing season.

To test our third hypothesis, whether changes in distance to the edge from 2023 to 2024 as a result of the right‐of‐way widening corresponded with a shift in growth, flowering, and seed production, we calculated the relative spatial shift of each individual plant Δ*d = d*_2024_–*d*_2023_ (where *d* is the distance of the individual plant to the edge) given that the edge shift was not homogeneous across the site. For each plant, we also calculated the corresponding changes in plant height, flowering, and reproductive output between the 2 years Δ*R = R*_2024_–*R*_2023_ (where *R* is the response variable: plant height, number of flowers, or number of seed capsules). To test for a predictive trend in growth, flowering, and seed capsule count in response to changes in distance to the edge, we used a simple linear model.

To identify potential complementary resources which could lead to an apparent edge effect, we utilized a one‐way ANOVA that tests for differences in soil characteristics between the forest, edge, and grassland areas. A principal component analysis (PCA) was conducted on all measured soil characteristics (nutrients, pH, LBC, OM, and CEC). Prior to analysis, all variables were scaled. An analysis of similarities (ANOSIM) was then used to test for significant differences in soil characteristics between areas. Soil moisture was analyzed for differences among areas (forest, edge, grassland) using a linear mixed‐effects model (LMM) with area as a fixed effect, sensor ID as a random effect to account for repeated measures, and daily average volumetric water content as the response variable. Diurnal air temperature maxima were compared using a separate LMM with area (forest, edge, grassland) as a fixed effect and date as a random effect to account for day‐to‐day variation in temperature across sites. These modeling choices reflect the repeated nature of soil moisture measurements at each sensor and the expectation that temperature may vary systematically by day across all areas. All data were analyzed using ‘R’ statistical software (v4.3.2; R Core Team 2023) using packages tidyverse (v2.0.0; Wickham et al. 2019), vegan (v2.6‐4; Oksanen et al. 2022), and propagate (v1.0‐6; Spiess 2018).

## 3 Results

**FIGURE 2:**
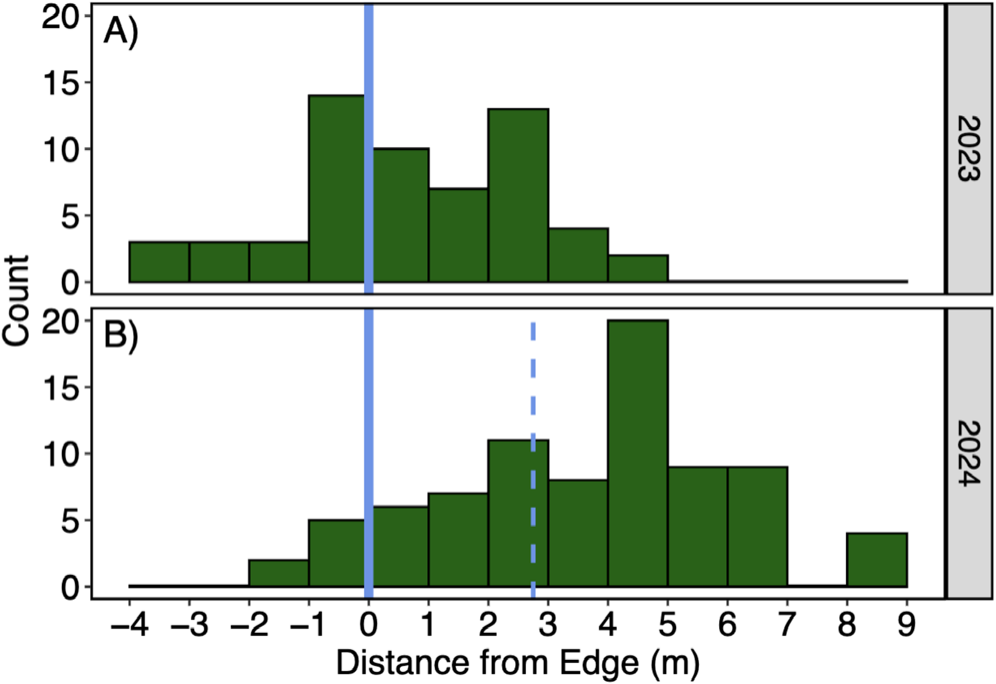
Count of *S. regia* individuals in relation to distance to edge in 2023 (A) and 2024 (B), with 0 (blue line) corresponding to the edge between forest and grassland areas. The dashed line in 2024 indicates the approximate location of the previous edge, shifted by 2.76 m on average. In total, there were 59 and 81 individuals in 2023 and 2024, respectively.

Individual *S. regia* plants were more abundant along the edge than the forested or grassland areas (Figure 2). In 2023, we found 9, 24, and 26 plants in the forest, edge, and grassland area, respectively. Accounting for the calculated areas, these counts translate to densities of 5, 20, and 11 plants per 100 m^2^ within the forest, edge, and grassland areas, respectively. Assuming a uniform distribution given a site‐wide density of 11 plants per 100 m^2^, expected counts were approximately 20, 13, and 26 in the forest, edge, and grassland areas, respectively. The observed distribution differed significantly from these expectations (*p* < 0.001), with fewer than expected plants in the forest and more than expected plants along the edge. In 2024 following the widening of the right‐of‐way, we found 2, 12, and 67 plants in the forest, edge, and grassland areas, respectively.

**FIGURE 3:**
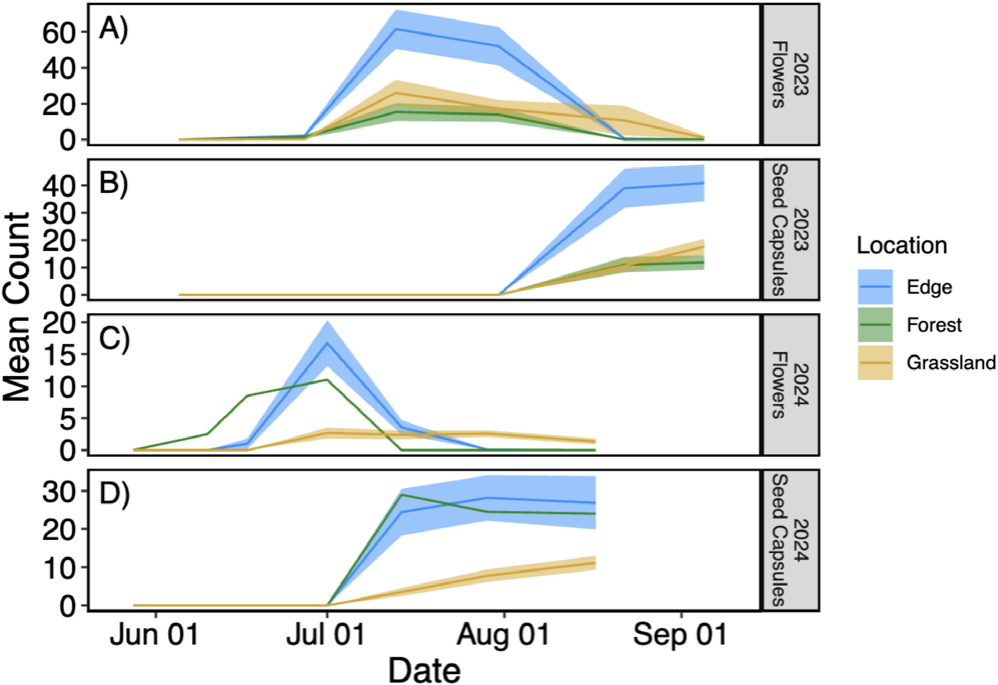
Flower (A, C) and seed capsule (B, D) count for the two sampling years 2023 (A, B) and 2024 (C, D). Lines indicate means and ribbons the standard error around the mean. Note that limited samples in 2024 in the forest did not allow for the calculation of the standard error.

Mean counts of flowers and seed capsules per plant were higher along the edge in both 2023 and 2024. At the observed peak flowering time in 2023 (July 13), we observed on average 15 flowers per plant in the forested area, 62 along the edge, and 26 within the grassland area (Figure 3A). Seed capsule counts began leveling off around mid‐August and reached on average 12, 41, and 18 in the forest, edge, and grassland areas, respectively (Figure 3B). Compared to 2023, observed peak flowering occurred earlier in 2024 (July 1) and was characterized by lower averages, with 11, 17, and 3 flowers per plant in the forest, edge, and grassland areas, respectively (Figure 3C). Seed capsule counts began leveling off in late July and reached on average 24, 27, and 11 in the forest, edge, and grassland areas, respectively (Figure 3D).

**FIGURE 4:**
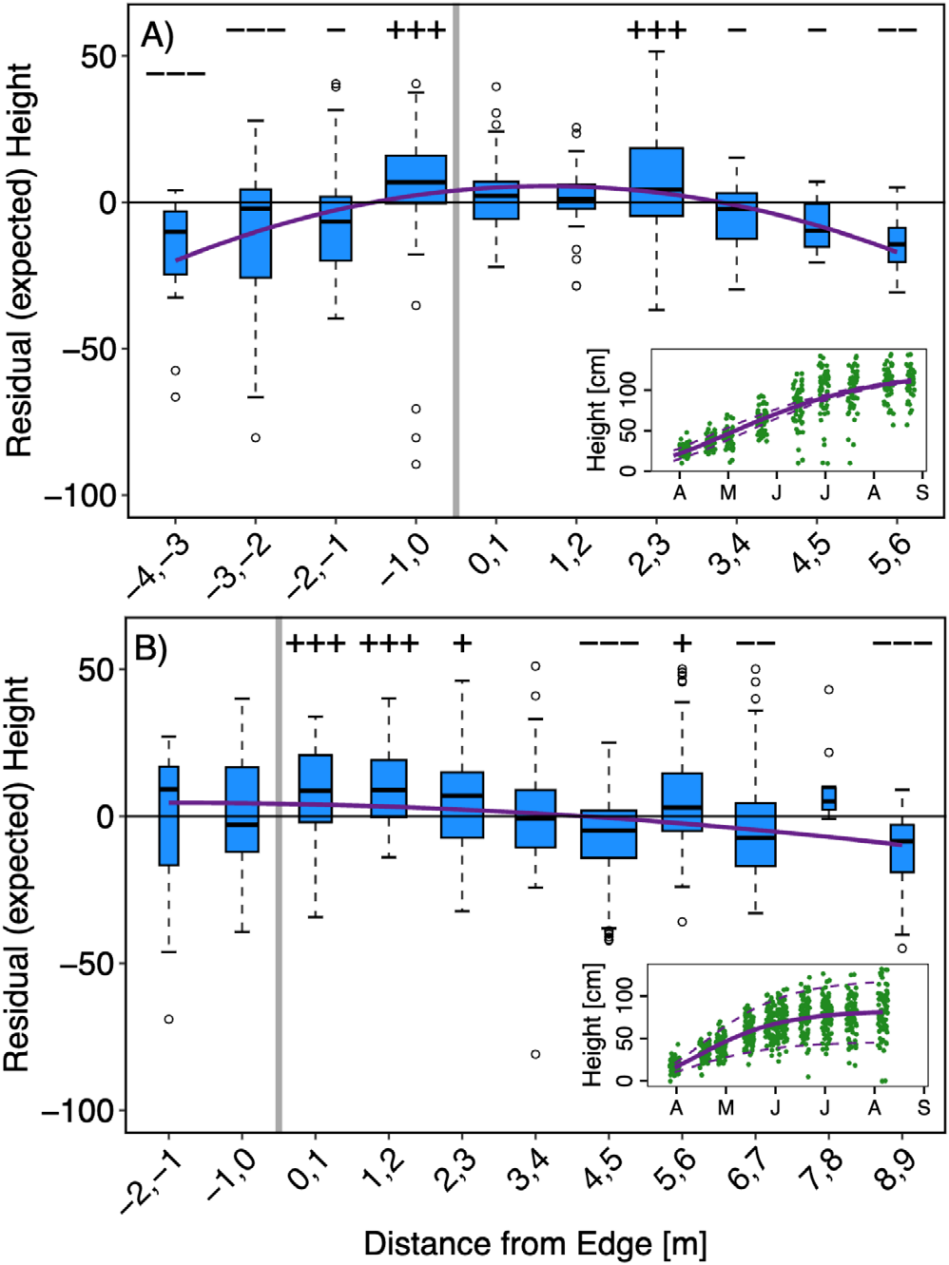
Residuals of plant height obtained from Gompertz growth model per each 1 m distance bin, with 0 corresponding to edge (gray line), in 2023 (A) and 2024 (B). A residual value of 0 notates expected height based on model output, positive values indicate greater than expected height, and negative values indicate height lower than expected. The width of the bars is proportional to the number of samples per bin. Solid purple lines indicate significant polynomial trends. Symbols above each bin denote statistical significance and direction of deviation from expected values: +++, ++, and + indicate significantly higher than expected values (*p* < 0.001, 0.01, 0.05, respectively), while −−−, −−, and − indicate significantly lower values. Insets: Observed plant height (in cm) over the sampling period (green dots), overlayed with modeled Gompertz curve (solid purple lines) and 95% confidence intervals (dashed purple lines).

Plant growth exhibited a significant asymptotic trend (*p* < 0.001), with a maximum average height of 126.0 cm (±5.2 cm SE) in 2023 (inset of Figure 4A), compared to a significantly lower height of 82.7 cm (±1.8 cm SE) in 2024 (inset of Figure 4B). Maximum growth rates, represented by the inflection points of the modeled growth curves, occurred on May 15 in 2023 and April 27 in 2024, continuing the trend of a 2‐week difference in timing between the two sampling years.

Residuals from the growth model exhibited a significant quadratic trend in both 2023 (*p* < 0.001; Figure 4A) and 2024 (*p* = 0.02; Figure 4B), with peaks aligning with the forest‐grassland edge in both years. In 2023, all plants located farther than 1 m into the forest and 3 m into the grassland exhibited significantly lower than expected growth, whereas plants growing between 1 m into the forest and 3 m into the grassland had heights that were as expected or significantly higher than expected (Figure 4A, Table 1). In 2024, following the widening of the right‐of‐way, plants growing within 3 m of the edge into the grassland were significantly taller than expected (Figure 4B, Table 1). Farther into the grassland, plant heights shifted towards significantly shorter than expected (4–5, 6–7, and 7–8 m), except at 5–6 m, where plants were again significantly taller than expected (refer to Table 1 for parameter estimates and *p*‐values).

**TABLE 1:** Estimated effects and *p*‐values from a linear model (no intercept) for plants growing at various locations (i.e., distance bins from the edge) along the forest to grassland gradient.

Location	Height effect (±SE)		Flowers effect (±SE)		Seed capsules effect (±SE)	
2023						
−4, −3	−16.86	(3.97)***	−0.59	(0.25)*	−0.55	(0.58)
−3, −2	−13.16	(2.94)***	−0.43	(0.20)*	−0.77	(0.41)
−2, −1	−6.63	(2.89)*	−0.30	(0.18)	−0.13	(0.33)
−1, 0	5.57	(1.50)***	−0.37	(0.09)***	0.62	(0.17)***
0, 1	1.90	(2.02)	−0.61	(0.12)***	−0.33	(0.22)
1, 2	1.27	(2.41)	−0.81	(0.15)***	−0.64	(0.29)*
2, 3	7.27	(1.70)***	−0.68	(0.10)***	−1.27	(0.20)***
3, 4	−4.91	(2.23)*	−0.52	(0.19)**	−1.12	(0.41)**
4, 5	−8.09	(3.68)*	−0.43	(0.23)	−0.23	(0.41)
5, 6	−13.80	(5.33)**	−0.27	(0.32)	0.02	(0.58)
2024						
−2, −1	−2.52	(3.80)	0.01	(0.17)	0.66	(0.31)*
−1, 0	0.95	(2.19)***	−0.19	(0.10)	0.85	(0.18)***
0, 1	8.10	(2.19)***	−0.44	(0.10)***	0.39	(0.18)*
1, 2	10.36	(2.03)*	−0.43	(0.09)***	−0.01	(0.16)
2, 3	4.31	(1.70)	−0.53	(0.08)***	−0.57	(0.14)***
3, 4	0.02	(1.79)	−0.51	(0.08)***	−0.68	(0.14)***
4, 5	−7.57	(1.29)***	−0.45	(0.06)***	−0.78	(0.10)***
5, 6	4.58	(1.79)*	−0.53	(0.08)***	−0.64	(0.14)***
6, 7	−5.16	(1.79)**	−0.61	(0.08)***	−1.12	(0.14)***
7, 8	9.83	(5.37)	−0.56	(0.24)***	−1.22	(0.43)**
8, 9	−11.14	(3.10)***	−0.54	(0.14)*	−1.22	(0.25)***

*Note:* ***, **, and * denote statistical significance (*p* < 0.001, 0.01, 0.05, respectively).

**FIGURE 5:**
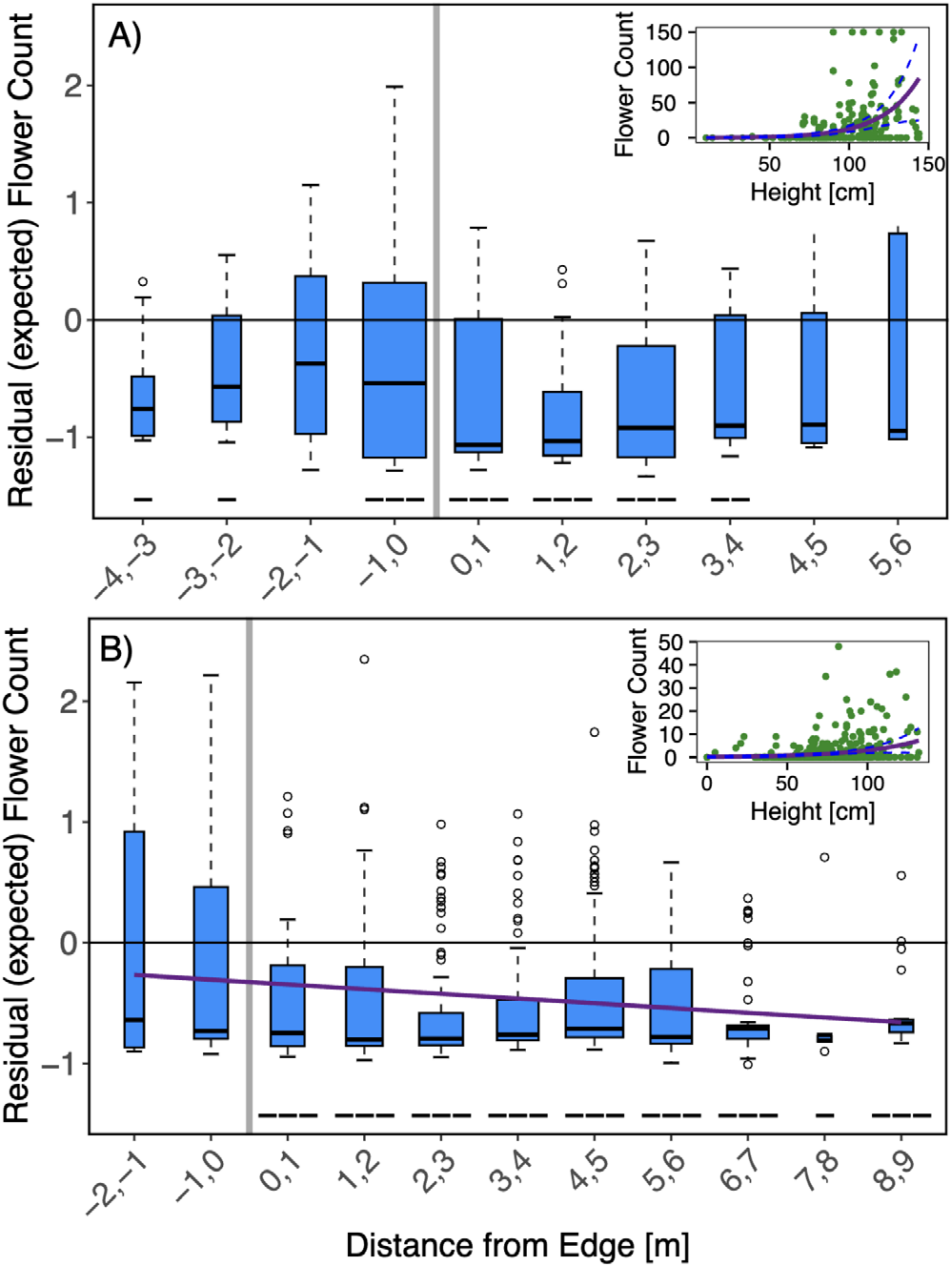
Residuals of flowering obtained from negative binomial model per each 1 m distance bin, with 0 corresponding to edge (gray line), in 2023 (A) and 2024 (B). A residual value of 0 notates expected flower count based on model output, positive values indicate greater than expected flower counts, and negative values indicate height lower than expected. The width of the bars is proportional to the number of samples per bin. Solid purple lines indicate significant polynomial trends. Symbols below each bin denote statistical significance and direction of deviation from expected values: +++, ++, and + indicate significantly higher than expected values (*p* < 0.001, 0.01, 0.05, respectively), while −−−, −−, and − indicate significantly lower values. Insets: Observed flower counts over the sampling period (green dots), overlayed with negative binomial generalized linear model (solid purple lines) and 95% confidence intervals (dashed purple lines).

**FIGURE 6:**
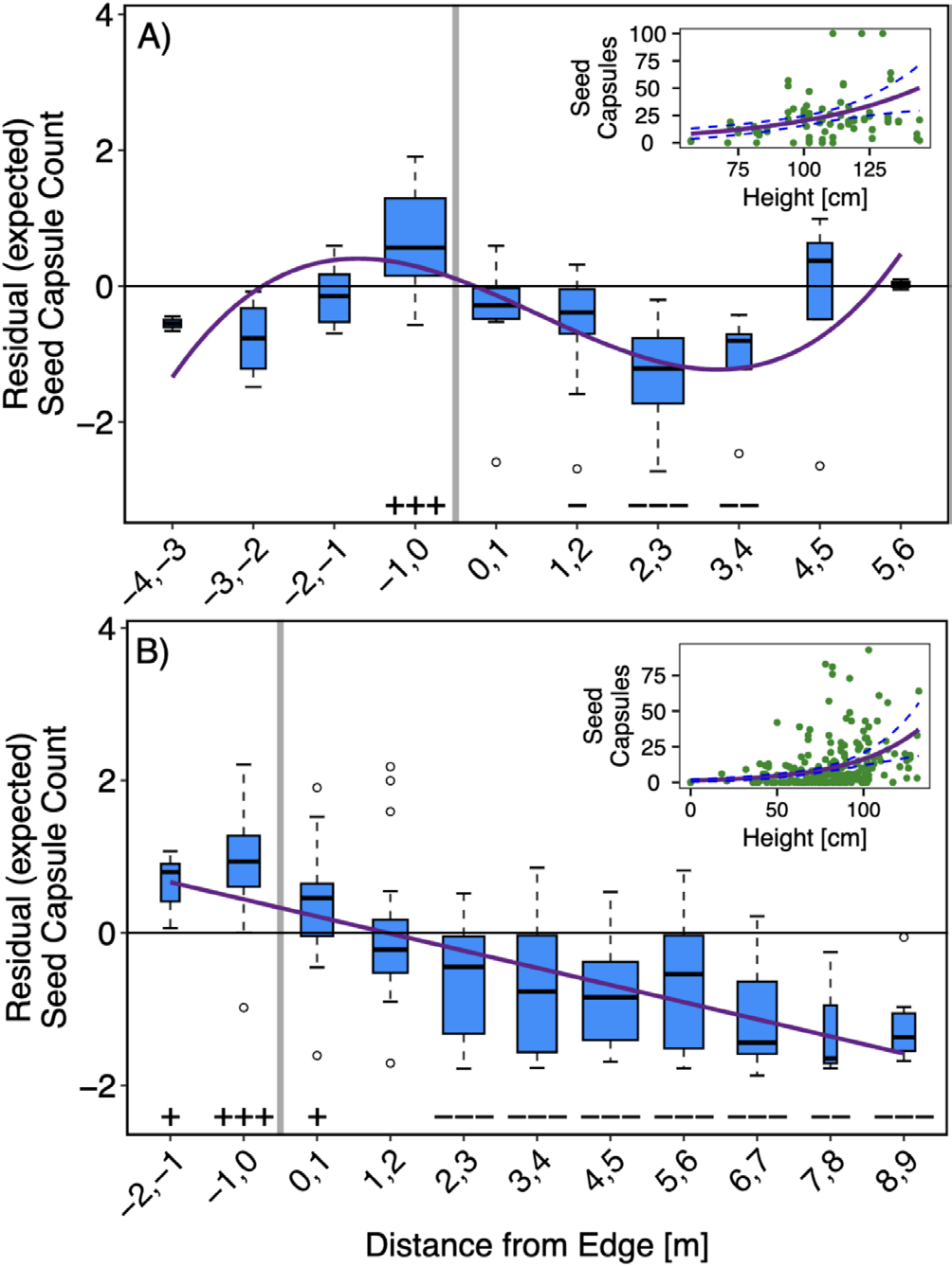
Residuals of reproductive output obtained from negative binomial model per each 1 m distance bin, with 0 corresponding to edge (gray line), in 2023 (A) and 2024 (B). A residual value of 0 notates expected seed capsule count based on model output, positive values indicate greater than expected seed capsule counts, and negative values indicate height lower than expected. The width of the bars is proportional to the number of samples per bin. Solid purple lines indicate significant polynomial trends. Symbols below each bin denote statistical significance and direction of deviation from expected values: +++, ++, and + indicate significantly higher than expected values (*p* < 0.001, 0.01, 0.05, respectively), while −−−, −−, and − indicate significantly lower values. Insets: Observed seed capsule counts over the sampling period (green dots), overlayed with negative binomial generalized linear model (solid purple lines) and 95% confidence intervals (dashed purple lines).

Plant height was a significant predictor of both flowering and seed production (*p* < 0.001 for all models; see insets Figures 5 and 6), with taller plants yielding more flowers and seed capsules. No significant trend was detected in flowering residuals in 2023 (Figure 5A), but values were significantly lower or as expected across the entire forest‐grassland gradient (Table 1). In 2024, flowering residuals exhibited a significant linear trend (*p* = 0.002), decreasing with greater distance into the grassland from the forest edge (Figure 5B), and all plants except those growing between 0 to 2 m into the forest exhibited lower than expected flowering. Residuals from the seed capsule model followed a significant cubic trend in 2023 (*p* = 0.007), with a peak located adjacent to the forest‐grassland edge (Figure 6A). Plants located along the edge up to 1 m into the forest had significantly higher than expected counts of seed capsules whereas plants growing between 1 to 4 m into the grassland produced significantly fewer than expected seed capsules (Table 1). In 2024, seed capsule residuals declined significantly with increasing distance from the edge into the grassland area (*p* < 0.001; Figure 6A). Edge‐adjacent plants, from 2 m into the forest to 1 m into the grassland, had significantly higher seed capsules whereas plants farther than 2 m into the grassland produced significantly fewer seed capsules than expected (Table 1).

**FIGURE 7:**
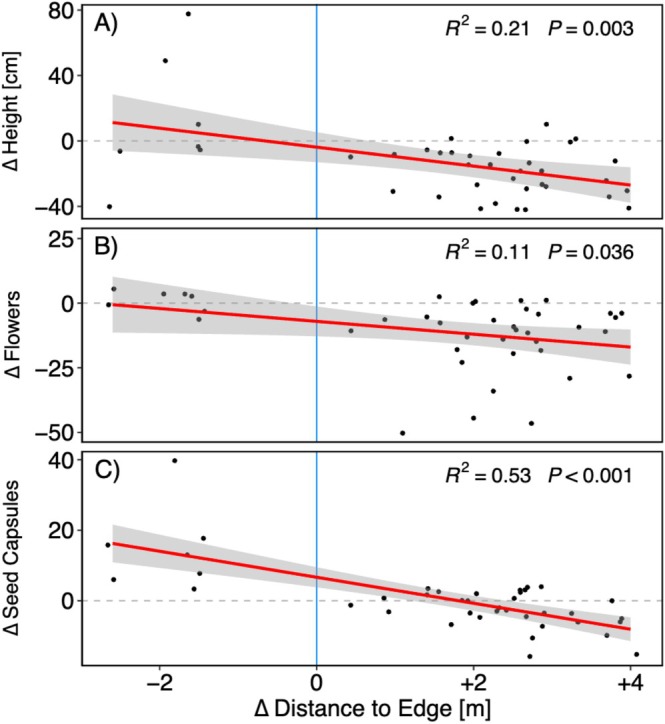
Changes in plant height (A), flower counts (B) and seed capsule counts (C) in response to changes to the distance from the edge between 2023 and 2024 (after the widening of the right of way). Each point represents one plant; only plants that were found and measured in both years were included in this analysis (*N* = 44). The red line indicates the fitted linear regression line, with the shaded area representing the standard error around the mean prediction. *R* ^2^ and *p*‐values from the linear model are provided within each plot. The blue line represents no change in distance to the edge; positive values denote an increase and negative values a decrease in distance to the edge.

Year‐to‐year changes in height, flowering, and seed capsule counts from plants measured in both years shows a clear linear trend (Figure 7) corresponding with the shift of the edge. Plants for which the distance to the edge decreased (i.e., plants previously in the forest) responded predominantly positively across all metrics, whereas increasing the distance to the edge resulted predominantly in reduction in growth, flowering, and seed capsule production.

Of the 19 soil metrics tested in 2023, only the concentrations of magnesium (Mg) and phosphorus (P) exhibited significant differences between the forest, edge, and grassland areas. The edge area had a significantly lower concentration of Mg (*p* = 0.02) and a significantly higher amount of P (*p* = 0.04) than either the forest or grassland areas (Table 2). ANOSIM detected no significant differences, indicating that although Mg and P differed significantly among zones, these differences did not translate into distinct overall soil profiles between the three areas.

**TABLE 2:** Results of soil composition testing including properties and extractable nutrients. Mean values and standard errors for forest, edge, and grassland areas are shown. Significant differences are denoted with an asterisk. Values with different letters are significantly different from one another. CEC, cation exchange capacity; LBC, lime buffer capacity; OM, organic matter.

	Forest (±SE)		Edge (±SE)		Grassland (±SE)	
Soil property						
LBC (ppm CaCO_3_ pH ^−1^)	526.50	(30.82)	782.67	(172.04)	449.20	(35.16)
pH	7.27	(0.17)	7.44	(0.17)	7.51	(0.18)
Base Saturation (%)	99.29	(0.65)	99.40	(0.60)	99.72	(0.28)
OM (%)	14.63	(1.29)	12.07	(1.38)	11.72	(0.38)
CEC (meq 100 g^−1^)	28.16	(4.74)	24.95	(3.76)	27.42	(4.17)
Soil nutrient (kg ha^−1^)						
Ca (Calcium)	12008.34	(2270.40)	11250.17	(1795.59)	12053.22	(2105.25)
Cd (Cadmium)	0.03	(0.01)	0.01	(0.00)	0.02	(0.01)
Cr (Chromium)	0.03	(0.02)	0.01	(0.00)	0.01	(0.00)
Cu (Copper)	0.35	(0.09)	0.38	(0.07)	0.37	(0.07)
Fe (Iron)	8.90	(1.54)	12.28	(2.57)	10.59	(2.18)
K (Potassium)	177.96	(11.00)	198.42	(26.53)	164.41	(9.71)
Mg* (Magnesium)	489.97	(32.88)	383.61	(28.07) ↓	433.40	(19.68)
Mn (Manganese)	91.17	(15.80)	91.91	(14.59)	63.76	(14.88)
Mo (Molybdenum)	0.09	(0.00)	0.08	(0.00)	0.08	(0.00)
Na (Sodium)	32.63	(6.76)	34.42	(3.64)	34.50	(5.66)
Ni (Nickle)	0.39	(0.09)	0.45	(0.08)	0.35	(0.08)
P* (Phosphorus)	34.20	(4.75)	64.43	(12.90) ↑	33.74	(8.07)
Pb (Lead)	0.79	(0.31)	1.06	(0.50)	0.81	(0.20)
Zn (Zinc)	22.04	(14.06)	4.95	(0.55)	7.44	(1.65)

*Note:* * denotes statistical significance (0.05).

**FIGURE 8:**
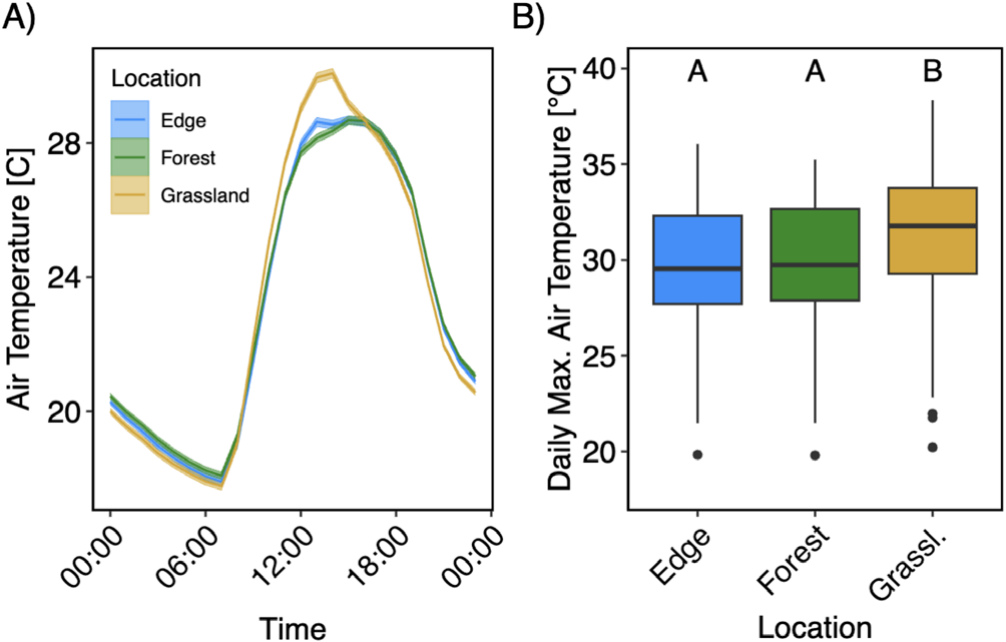
Diurnal mean air temperatures (A) and boxplot of mean maximum daily temperatures (B) in forest, edge, and grassland areas from July 27 to October 4, 2024. Shaded areas in (A) represent standard errors of the daily mean across sensors within each location. Letters in (B) indicate significant differences between locations based on post hoc comparisons (Tukey‐adjusted *p* < 0.05).

Soil moisture and local air temperature data from 2024 suggest significant microclimate variation between the three areas. Diurnal air temperature patterns show notably higher temperatures in the grassland around noon (Figure 8A). This was reflected in significantly higher daily maximum temperatures in the grassland compared to both the forest and edge (*p* < 0.001; Figure 8B), with the model estimating identical maxima for the forest and edge areas (29.7°C; 95% CI: 28.8°C–30.6°C) and higher values in the grassland (31.2°C; 95% CI: 30.3°C–32.1°C).

**FIGURE 9:**
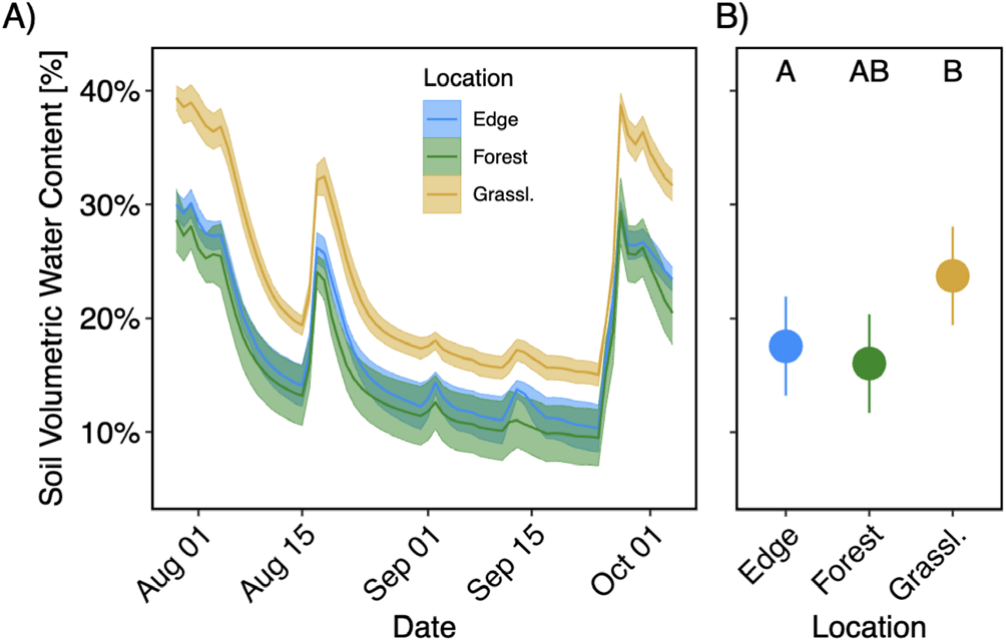
Daily average soil volumetric water content (VWC; A) and estimated marginal means (±95% CI) from a linear mixed‐effects model (B) in forest, edge, and grassland areas from July 27 to October 4, 2024. Shaded areas in (A) represent standard errors of the daily mean across sensors within each location. Model predictions in (B) account for repeated measures at the sensor level. Letters indicate statistically significant groupings from post hoc comparisons (Tukey‐adjusted *p* < 0.05).

Model‐estimated daily average soil volumetric water content was 16.0% (95% CI: 11.7%–20.4%) in the forest area, 17.6% (95% CI: 13.2%–21.9%) along the edge, and 23.7% (95% CI: 19.4%–28.1%) in the grassland area. Grassland soils had significantly higher moisture than forest (*p* = 0.0496), while soil moisture along the edge was not significantly different from either location (Figure 9B).

## 4 Discussion

Our first hypothesis, that there will be a higher abundance of *S. regia* individuals in close proximity to the edge between forest and grassland, was supported by our data. These findings align with the framework outlined by Ries et al. (2004), that a species density and abundance will increase along edge boundaries in which there are advantageous resources present on either side. Erdős et al. (2019) provide evidence that edges maintain a unique species composition in which several grassland species appear to relate themselves more to edge areas than grassland interiors. Interestingly, the same study observed an increase in species richness along north‐facing edges but not in south‐facing, suggesting that edge orientation may also play a role in influencing plant species and communities. The edge at our study site is oriented such that it receives shading from the west. Chen et al. (1993) found that western‐shaded edges maintain significantly higher soil moisture and experience reduced afternoon temperatures due to earlier shading, in contrast to eastern edges that receive intense solar radiation later in the day. Although soil moisture at our site was highest in the open grassland, likely because of a slight topographic depression, daily maximum air temperatures were significantly lower along the edge and within the forest. This thermal buffering at the edge may be particularly important for the survival of *S. regia* , which has a large taproot and may be more sensitive to heat stress than to transient drought conditions.

Our second hypothesis, that plants occurring along the edge will exhibit greater height, increased flowering, and higher rates of successful seed capsule production, was supported by our findings. Significant positive trends corresponding to the edge were found in both sampling years for growth, flowering, and seed capsule production, with the exception of flowering in 2023. Apart from reported occurrences in Jackson County in northern Florida, Georgia marks the southern boundary of *S. regia's* native range in the US (NatureServe 2025), a region characterized by high summer temperatures. The limiting impact of environmental stress on plant growth is well documented (Ramdhani et al. 2016; Li et al. 2020; Zhang et al. 2020). Canada goldenrod ( *Solidago canadensis* ), another C_3_ forb native to Georgia (US), has been shown to exhibit decreased growth in response to periods of heat stress (Wang et al. 2016), displaying significantly reduced aboveground production compared to plants not subjected to warming. In our study, *S. regia* plants that were located along the edge and in the forest were shielded from intense afternoon heat by the western directional shading, likely reducing heat stress and allowing for higher resource allocation towards growth.

Our models align with previous work on *S. regia* showing that flower and seed capsule production are positively associated with increased plant size (E. Menges 1988). In our study, the earlier estimated peak growth rate in 2024 may help explain the overall lower plant heights observed in that year, which in turn likely contributed to the reduced overall flower and seed capsule production. These differences are potentially the result of interannual local climate variation, which can directly affect the timing of vegetative growth and the allocation of resources to reproduction.

The relationship between vegetative growth, reproductive output, and overall allocation of resources is a core concept in plant life history strategies (Weiner 2004; Weiner et al. 2009). Evidence generally indicates that in perennial species, larger individuals and individuals growing in low‐stress environments allocate more resources to reproductive output (Sun and Frelich 2011; Du and Qi 2010; Weiner et al. 2009; Liu and Pennings 2019). Increased investment in height may also serve to improve pollinator visibility, as animal‐pollinated plants often grow taller and produce larger floral displays to attract pollinators (Hernández‐Villa et al. 2020). Because *S. regia* is pollinated by hummingbirds, taller individuals capable of allocating resources to more conspicuous floral displays are more likely to attract visits (Dudash et al. 2011), resulting in higher production of viable seeds.

The observed higher abundance, increased growth, flowering, and seed capsule production of *S. regia* in edge‐adjacent locations in our study is likely the result of multiple interacting factors, both biotic and abiotic. One main driver is likely the lower observed daily maximum temperature along the edge. This aligns with findings by Tsogtsaikhan et al. (2025), who report higher reproductive investment under lower climate variability (i.e., more stable climatic conditions) and decreased allocation to reproductive structures under climatic stresses for 22 *Artemisia* species. More broadly, canopy structure is known to generate strong microclimatic gradients, influencing temperature extremes, light availability, and humidity across forest–grassland transitions (Gril et al. 2025). Such microclimatic variation is increasingly recognized as important for mediating species responses to climate stress and shaping plant distributions (Hylander et al. 2022).

In addition to climate variability, plants in terrestrial ecosystems are often limited by nutrient availability (Fay et al. 2015), with nitrogen (N) and phosphorus (P) being the most common limiting nutrients in grassland systems (Fisher et al. 2012). The significantly higher phosphorus concentrations along the edge, potentially resulting from increased bird activity and associated guano deposition, could further explain the greater abundance and increased vigor of *S. regia* in these locations, as phosphorus is essential for synthesizing ATP, NADPH, nucleic acids, sugar phosphates, and phospholipids, which are critical to photosynthesis and overall plant metabolism (Carstensen et al. 2018; Hammond and White 2007; Lambers 2022). However, more in‐depth examination of physiological processes and stress levels in *S. regia* would be required to confirm if P acts as a limiting nutrient to this species at this site.

Interspecific competition is another important factor to consider when examining the apparent edge‐favoring behavior of *S. regia* at this site. Anecdotal observations suggest that other co‐occurring large native grassland species, including *Rudbeckia triloba, Silphium terebinthinaceum*, and *Solidago gigantea* , were predominantly found in the open grassland area. Competitive interactions can be strongly influenced by environmental factors, including partial shading, as demonstrated in a calcareous grassland by Corcket et al. (2003). Consequently, species interactions along habitat edges can be expected to differ from those in open grassland or forest areas, potentially favoring *S. regia* . Competition for light also plays a key role in promoting both above‐ and below‐ground productivity of grassland plant species, creating a positive correlation between species richness and plant production (Zhang et al. 2014). Numerous studies have provided evidence that edges between habitats support unique species compositions and greater diversity (Erdős et al. 2013, 2019; Ohara and Ushimaru 2015; Burst et al. 2017), and that biodiversity enhances facilitative interactions in grassland systems (Mahaut et al. 2020; Cardinale et al. 2012; Tilman et al. 2001). For example, increased diversity at habitat edges may reduce competitive dominance and promote coexistence through niche partitioning and complementary resource use (Tilman et al. 2001; Cardinale et al. 2012). Additionally, habitat edges can support higher pollinator abundance and visitation due to increased floral resources and landscape heterogeneity (Ren et al. 2023). Together, our observations suggest that *S. regia* is either limited by stronger competition in the open grassland and/or benefits from facilitative interactions along the edge. Future research should include a complete floristic survey as well as common garden experiments and additional study sites to test for potential differences in species interactions under varying light conditions.

The shift in peak values between 2023 and 2024, coinciding closely with the change in the physical location of the edge, provides strong support for our final hypothesis. Deviating from this trend is a single localized peak in plant height at 5–6 m in 2024, corresponding to the location of greater‐than‐expected plant height at 2–3 m in 2023 and thus aligning with the 2.76 m shift. We interpret this pattern as evidence that individuals with greater stored energy reserves in their taproots were able to maintain enhanced growth into the subsequent year, even as the edge position shifted. Overall, our data lend weight to the proposed edge effect in *S. regia* by offering experimental evidence rather than relying solely on observational data. Considering a changing climate and the location at the southern boundary of its geographical range, it may be necessary for a predominantly Midwestern grassland species like *S. regia* to either migrate to more suitable regions or rely on microrefugia conditions to persist in Georgia. Microrefugia often maintain systematically colder climates, especially during the hottest months, and can support unique species compositions (Finocchiaro et al. 2023). Globally, extirpations from previously suitable locations as well as migrations and complex range shifts, primarily to cooler regions from original warmer ranges, have been widely documented (Angert et al. 2011; Anderson and Wadgymar 2020; Rubenstein et al. 2023). Such dynamics can result in “trailing” range edges, where populations contract away from newly unsuitable parts of their range (Sheth and Angert 2018). Many species are also adjusting phenologically, emerging or reproducing earlier in response to shortened or milder winters (Cook et al. 2012; Wadgymar et al. 2018). As noted by Kull (2020), the drivers of *S. regia* population loss are likely complex and interactive. Decline of suitable habitat for the species, coupled with a lack of natural fire regimes and increasing stress likely all play a role in this species seeking refuge along a forest‐grassland edge. Our study focuses on the immediate response of established individuals of *S. regia* , and the second year of observation likely represents a transitional period following the shift in edge location, during which the plant community had not yet stabilized. The absence of individuals further into the forest may reflect limited dispersal distances and the time required for successful establishment, suggesting that longer‐term monitoring will be necessary to capture potential redistribution and migration of this species.

Lastly, proximity to the trees along an edge could provide protection from anthropogenic disturbances such as trampling, ATV traffic, or mowing, as well as natural disturbances such as deer herbivory. However, we did not observe any evidence of human‐induced disturbances of any kind at this site, and damage from herbivory was rarely observed and seemingly random across plants. Continued yearly monitoring of this site may be beneficial to examine whether observed trends are consistent across years.

In summary, our results provide evidence that on the southern range of its distribution, *S. regia* benefits from taking refuge along forest‐grassland edges. We conclude that edge habitat, functioning as both a structural boundary and a climatic and competitive refuge, may be a key requirement to prevent further decline of *S. regia* in Georgia. Future conservation efforts along the species' southern range should take the presence of partial shade into account to maximize survival and vigor of the population.

## Author Contributions

**Megan Nicole Brooks:** conceptualization (equal), formal analysis (equal), investigation (equal), writing – original draft (lead). **Adamina Bilbrey:** formal analysis (equal), investigation (equal), writing – review and editing (equal). **Heather Bowman Cutway:** supervision (supporting), writing – review and editing (equal). **Nicholas Green:** formal analysis (equal), supervision (supporting), writing – review and editing (equal). **Mario Bretfeld:** conceptualization (equal), data curation (equal), formal analysis (equal), supervision (equal), writing – review and editing (equal).

## Funding

This work was supported by Birla Carbon, 2024 Scholarship and Georgia Botanical Society, 2022 Marie Mellinger Field Botany Research Award.

## Conflicts of Interest

The authors declare no conflicts of interest.

## Data Availability

The data that support the findings of the study are available at https://doi.org/10.6084/m9.figshare.31847203.
